# The Suckling Rat as a Model for Immunonutrition Studies in Early Life

**DOI:** 10.1155/2012/537310

**Published:** 2012-07-31

**Authors:** Francisco J. Pérez-Cano, Àngels Franch, Cristina Castellote, Margarida Castell

**Affiliations:** ^1^Department of Physiology, Faculty of Pharmacy, University of Barcelona, Av. Joan XXIII, 08028 Barcelona, Spain; ^2^Institut de Recerca en Nutrició i Seguretat Alimentària (INSA-UB), 08028 Barcelona, Spain

## Abstract

Diet plays a crucial role in maintaining optimal immune function. Research demonstrates the immunomodulatory properties and mechanisms of particular nutrients; however, these aspects are studied less in early life, when diet may exert an important role in the immune development of the neonate. Besides the limited data from epidemiological and human interventional trials in early life, animal models hold the key to increase the current knowledge about this interaction in this particular period. This paper reports the potential of the suckling rat as a model for immunonutrition studies in early life. In particular, it describes the main changes in the systemic and mucosal immune system development during rat suckling and allows some of these elements to be established as target biomarkers for studying the influence of particular nutrients. Different approaches to evaluate these immune effects, including the manipulation of the maternal diet during gestation and/or lactation or feeding the nutrient directly to the pups, are also described in detail. In summary, this paper provides investigators with useful tools for better designing experimental approaches focused on nutrition in early life for programming and immune development by using the suckling rat as a model.

## 1. Introduction

The impact of nutrition on neonatal growth and early-life physiology is essential, not only because this is a critical stage of development and adaptation but also because it has a potentially long-lasting impact. In this sense, human epidemiologic data have indicated that prenatal and early postnatal nutrition modulates developing functions and influences adult susceptibility to diet-related chronic diseases. This lasting effect until adulthood is now referred to as “imprinting” or “programming” [1, 2]. Focusing on the immune function during early life, the relationship between nutrition and gut microbiota, mucosal homeostasis and immune programming has been reviewed [3–5]. In order to confirm epidemiologic associations, dietary interventions in human neonates have been performed; however, there are several limitations, such as ethical concerns and methodological aspects (type of samples, study design—preventive *versus* curative—in health and disease, dosage, genetic heterogeneity, etc.) [6]. In addition, when intestinal immune function is examined, tissue samples can only be obtained in a hospital environment from patients with prescribed intestinal biopsies, limiting, therefore, the number of samples that can be analyzed. Animal models have the advantage of allowing invasive tissue sampling to assess nutrient status and easily monitor compliance with the dietary protocols [7]. In fact, animal studies are needed to focus and direct further studies conducted in humans. In this sense, there is no doubt about how research using animals as experimental models has contributed to increasing the current knowledge about the interaction between diet and physiology, and more specifically about the immune system.

In this context, immunonutrition studies using animal models have been able to elucidate not only the effect of particular nutrients or diets on immune functions but also the precise mechanisms involved in these responses [8]. These studies have usually been performed on adult animals through the consumption of enriched diets or by direct administration of dietary supplements (oral gavage) such as vitamins, minerals, polyunsaturated fatty acids (PUFAs), fiber, probiotics, prebiotics, and other ingredients. However, the impact of nutrients on the developing immune system in animals during gestation or early life has been studied less. Thus, the purpose of this paper is to describe the potential of the suckling rat as a model for immunonutrition studies in early life and, more specifically, to provide tools for the investigators for better designing experimental approaches focused on the importance of nutrition in early life for programming and immune development.

## 2. Animal Models for Early Nutritional Studies

The importance of developing animal models to examine the nutritional effects on human health and disease led to the organization of the symposium about Appropriate Animal Models for Nutritional Research in Health and Disease, celebrated in Washington in 2007 in the context of the “Experimental Biology Meeting”. The symposium was intended to provide both conceptual and technical guidance to help expand the interactions between human and animal nutritional scientists [7, 9].

While animal models for the study of human neonatal nutrition include mice, rats, rabbits, guinea pigs, dogs, pigs, and nonhuman primates [6], the species that have provided the most useful nutritional information are rodents (mainly mice and rats) and pigs, particularly in terms of the interaction between nutrients and the immune system. However, there are several aspects that must be considered when choosing the most appropriate animal model for a study and it should be based on the desirability for a specific intervention and evaluation procedure. In this sense, Table 1 compares some physiological characteristics and practical information that should be taken into account regarding these three species.

Among the factors included in Table 1, the length of life periods is important: gestation, suckling, puberty, and life expectancy, all of which are shorter in rodents than in the pig model, which facilitates the nutritional intervention along one or several of these developmental stages [6, 10, 11]. Other factors that influence the overall relative desirability of mice, rats, and pigs are the cost, which includes not only the price of a particular animal but also its housing, the availability or the ease with which animals are obtained from the supplier, and the manageability, comprising the housing requirements, the time needed to take care of the animals and the ease of handling [12, 13].

Based on these criteria, rodents are generally the most economical models; they are more easily available and managed in the laboratory than larger animals [12]. Moreover, rodents are well-characterized models and provide many particular options, such as different strains, and knockout or transgenic animals, among others, which help in the process of resembling pathologies. However, the rodent models have some limitations regarding the extrapolation of the results to humans, such as differences in food intake and energy expenditure for body size, lifespan, and morphology and other physiological aspects [16]. If newborn mice and rats are compared as neonatal models, the diminutive size of mice pups constitutes the main obstacle to their choice, especially if the nutritional intervention has to start from the first day of suckling by artificial rearing.

Regardless of the limitations associated with large animals—such as long length periods, relative cost, availability, and manageability in a laboratory setting—in the symposium about Appropriate Animal Models for Nutritional Research in Health and Disease, the benefits of large-animal research models for nutrition were admitted because of their greater physiological similarity to humans than rodents [9]. In this regard, in recent years the young pig has come into particular prominence as an animal model for nutritional research due to its much closer resemblance to human physiology [7, 14, 16].

It should be taken into account that there are vast differences in the bioavailability of certain nutrients between animal models and humans. There are well-documented differences in how they use, metabolize, and excrete nutrients [7, 17]. For example, the selection of the animal model for studying vitamin A supplementation is crucial because the carotenoids are very differently absorbed depending on the selected model [7].

As no one technique or animal model is perfect, and as different methodological approaches are used to complement each other to increase the understanding of a particular question, it would be very helpful to use different animal models to address the issues of interspecies differences and to better predict what might happen in humans. Overall, although at present both rodents and pigs are used to study nutrient interactions with the immune system in early life; on the basis of our own experience [15], we will focus this paper on the suckling rat as a suitable model for immunonutrition studies.

## 3. Immune System Development in the Rat

In rats, as in humans and other rodents, the ontogeny of the immune system starts in the embryo and continues during fetal life, but it actually finishes several years after birth [18]. In general, the immune system in mammals is not fully functional at birth and develops later. This is due, in part, to the low exposure to antigen before birth. The ontogeny of immune system in rats is parallel to that in mice and it is delayed compared with humans probably due to their shorter gestation period. Thus, although the lymphoid architecture forms prenatally in humans and mostly postnatally in rodents, it seems that they both develop via similar schemes [19].

### 3.1. Ontogeny of the Systemic Immune System

Data about immune system development in rat fetus are focused on thymus. It has been reported that the number of thymic cells is very low before day 14 of fetal life, but increases exponentially during the last week gestation and from birth to reach adulthood [20]. After birth, by the beginning of the second week of rat life, a sudden increase in TCR*αβ*+ cells and a decrease in CD4−CD8− cells appear in the thymus [20]. A deeper study, performed in mice during fetal life, shows that the first cells populating the thymus rapidly differentiate and give rise to both cortical and medullar lymphoid populations of thymus; a second generation of precursors enters the thymus during the second half of fetal period and gives rise to a second generation of thymocytes which grows exponentially and replaces the first one at the end of the second week after birth [21]. The ontogeny of thymus in rodents is similar to that reported in humans. The embryonic thymus in humans appears around 6 weeks gestation [22], and later, T-lymphocyte differentiation begins. At week 10, the thymus has differentiated cortical and medullar zones with T cells and, at week 13, T cells colonize the fetal liver, spleen, and bone marrow [23].

**Figure 1:**
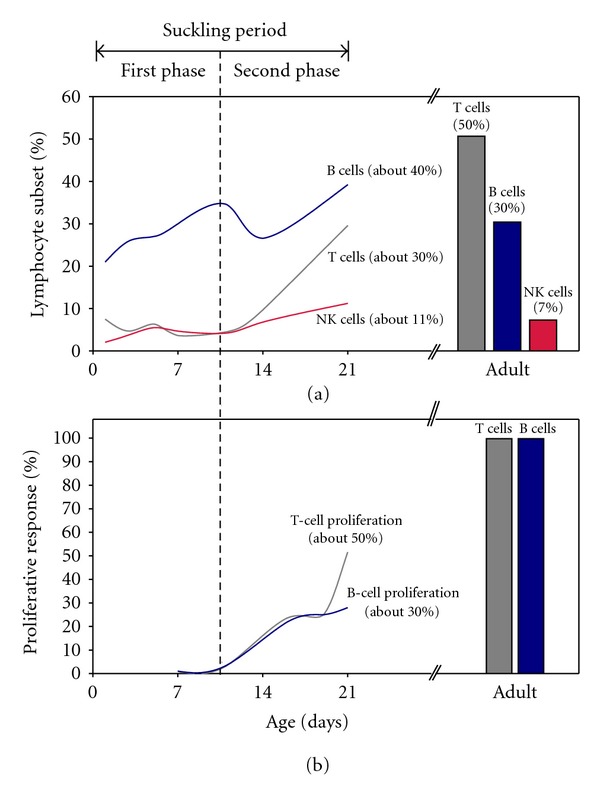
Developmental pattern of the systemic immune system in rats from suckling to adult age. Spleens of Lewis rats were obtained at several time points during suckling and, after mechanical spleen disruption, splenocytes were isolated by density gradient. Cells were stained by fluorochrome-conjugated monoclonal antibodies directed against several lymphocyte surface molecules (CD45RA for B cells, TCR*αβ* for T cells and NKR-P1A for NK cells). The percentage of each subset was established by flow cytometry analysis. Other splenocytes were incubated in presence of Concanavalin A or pokeweed mitogen over 72 h and proliferating cells were identified by means of a cell proliferation assay [24]. (a) Main spleen lymphocyte subsets during suckling in Lewis rats (expressed as a percentage of total spleen lymphocytes). (b) Proliferative response of neonatal spleen cells with concanavalin A (T-cell proliferation) and pokeweed mitogen (B-cell proliferation) in comparison with adult Lewis rats (which is considered as 100%). Results are estimated from data obtained from 1, 3, 5, 7, 11, 14, and 21-day-old rats (modified from [24]).

To establish the immune development in rats after birth, we have studied the appearance of lymphocyte subsets in spleen of newborn Lewis rats during suckling by means of immunofluorescence and flow cytometry [24]. We have observed that T lymphocytes in neonatal rat spleen are found in very low proportions during the first two weeks of life [24]. In rats, B lymphocytes constitute the earliest occurring population in the spleen after birth (Figure 1(a)), and during the suckling period these cells present an immature phenotype characterized by low surface IgM expression [24]. Similarly, in mice, immature B cells are also described in the neonatal spleen [25]. Data from humans report that B cells appear in the fetal spleen together with liver, bone marrow, and lymph nodes at 7-8 weeks of gestation, and these B cells possess an immature phenotype with no surface immunoglobulins but cytoplasmic IgM [26].

Studies about phenotypical lymphocyte composition performed by flow cytometry analysis of the spleen cell suspensions during the rat suckling period [24] have allowed two phases to be defined: early neonatal life (first half) and late suckling period (second half) (Figure 1(a)). Early neonatal life in the spleen (first phase) is characterized by a low proportion of CD4+ cells and CD8+ cells with immature phenotype [24]. In the second phase of the suckling period (i.e., 10–21 days), the number of CD4+ and CD8+ cells increases, and lymphocytes bearing CD3, TCR*αβ*, CD5, and CD2 molecules appear in the spleen (as can be seen in the T-cell pattern of Figure 1(a)) [24]. NK cells are present in liver and spleen from suckling mice [27] and in the spleen from suckling rats (Figure 1(a)) in a similar proportion to that found in adult rats [24], suggesting a key role of these cells in the defenses of newborn rodents. Moreover, NKT cells are present in the newborn rat spleens where they may exert some regulatory function and play a role in peripheral tolerance. In humans, NK cells are predominant during early infancy to early childhood [28], but their activity is reduced in newborns in comparison with children and adults [29].

Rat neonatal T and B immune responses are low at birth, less competent and functionally deficient compared with adult animals, as observed in mice [32]. Similarly, spleen lymphocytes from rat offspring present a very low proliferative response, increasing at suckling although it does not reach adult ability at weaning (Figure 1(b)) [24]. This feature is similar to human's newborns: the immunoproliferative response of neonatal T lymphocytes to mitogens is lower than in children and adults [33]. On the other hand, neonatal antibody response in rats is slower and exhibits lower average affinity and reduced diversity compared with adult counterparts [24, 33–35]. Sera immunoglobulin (Ig) G and IgM are already detected in 5-day-old rats and increase progressively during suckling. Similar to humans, IgA is the last antibody isotype to appear in rat sera [24].

Mice neonates, like human newborns, develop Th cell responses biased to Th2 [25, 36]. Th1 immune response is compromised with a deficient production of Th1 cytokines (IFN*γ*) and hyporesponsiveness of neonatal macrophages to this cytokine [37]. Similarly, rats primed during the first week of life produce antibodies depending of Th2-responses but, in contrast to adult rats, failed to develop Th1-dependent antibodies [38]. Th2 polarization in the fetus plays a physiological role because otherwise Th1 response could induce fetal damage. This Th1 response would involve excessive IFN*γ* production that is not only deleterious to the placenta integrity, but also the major cause of fetal loss [39, 40].

### 3.2. Ontogeny of the Intestinal Immune System

The mucosal immune system, also known as mucosa-associated lymphoid tissue (MALT), is more complex than its systemic counterpart and it includes the gut-associated lymphoid tissue (GALT), the nasopharynx-associated lymphoid tissue (NALT) and the bronchus-associated lymphoid tissue (BALT), among others [41, 42]. The lymphoid cell distribution in various compartments of the MALT is different among some animal species, and, for example, rodents and humans clearly differ in the anatomy of the NALT [42]. However, bearing in mind that the intestine represents the main compartment reached by dietary immunomodulatory compounds, GALT should be the focus of attention here. In this sense, the GALT anatomy of rats is more similar to humans than other species because they both share, besides the classical structures that are mentioned below, lymphocyte-filled villi, which are absent in pigs and mice; moreover, these latter species have lymphoid structures lacking in humans such as continuous ileal Peyer's patches and cryptopatches, respectively [41].

Peyer's patches (PPs), lymphoid aggregates in the small intestine, constitute the inductive site of intestinal immune response. At the end of rat fetal life, on day 18 gestation, PP are visible, although as aggregates without T or B cells [43]. At birth, in the PP there are only a few T cells and with age this proportion increases while IgM+ cells also appear. On day 12 of life, neonatal PPs are structurally similar, but without germinal centers, to those in adult rats, although of a smaller size [44]. Thus, the number of cells within the PP is of about 0.5 × 10^6^ at the end of suckling and keeps increasing through early life: 1.2 × 10^6^ (day 28), 2.0 × 10^6^ (day 35) and 2.5 × 10^6^ (day 42) [45]. With respect to T-cell development, on day 21, 15% of PP cells are CD5+, 12% are CD4+ and only 4% are CD8+ [45]. PP development in rats is rather slower than that in humans. It has been described that, at four months gestation, B cells and T cells appear in human fetal PP although there is no evidence of germinal centre formation [46]. After birth, however, they develop rapidly due to stimulation from luminal antigens [47] and the number of PP increases from about 60 at birth to over 200 by 12–14 years [48].

**Figure 2:**
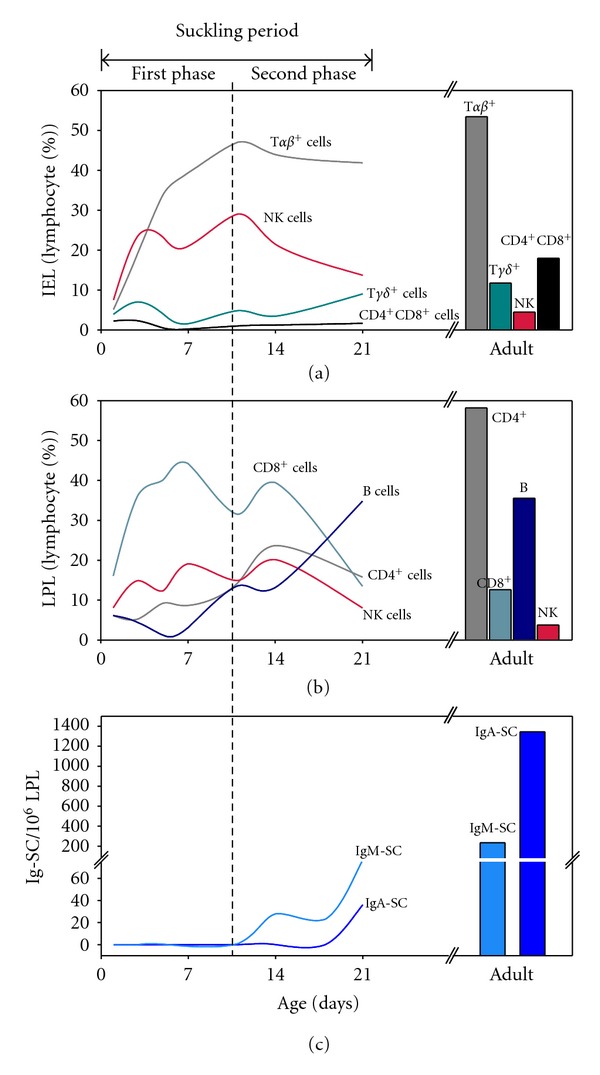
Pattern of maturation of the main effector lymphocyte populations in the rat small intestine during suckling. Results were estimated from data obtained from 1, 3, 5, 7, 11, 14, and 21-day-old rats. (a) Relative proportions of intraepithelial lymphocytes (IELs) in suckling and adult Lewis rats. Intestinal IEL suspensions were obtained by incubations with DTT, EDTA and medium, and subsequent purification with 44/67.5% Percoll. Immunofluorescence staining with anti-rat antibodies to CD4, CD8*α*, TCR*αβ*, TCR*γδ*, and NKR-P1A was then applied. Flow cytometry analysis allowed establishing the percentage of a particular IEL subset with respect to the total number of IEL [30]. (b) Relative proportions of lamina propria lymphocytes (LPLs) in suckling and adult Lewis rats. LPLs suspensions were obtained after removing IELs, by digestion with collagenase, and purified with 44/67.5% Percoll. LPL were then stained with fluorescence-conjugated antibodies to CD4, CD8*α*, CD45RA, and NKR-P1A. Analyses were performed by flow cytometry and cells were expressed as the percentage of positive cells with respect to total LPL [31]. (c) Number of IgM- and IgA-secreting cells (SC) in 10^6^ cells from small intestine lamina propria in suckling and adult Lewis rats. LPL suspensions were obtained after removing IELs by digestion with collagenase-dispase. Thereafter, serial dilutions of LPL were incubated in anti-rat IgM or IgA-coated plates. Biotin-conjugated anti-rat IgA or IgM, extravidin-peroxidase and colorimetric substrate allowed enumerating spots that corresponded to each secreting cell [31].

With regard to the development of intraepithelial lymphocytes (IELs), which are lymphocytes found in the epithelial layer of the intestinal mucosa, we have isolated and characterized by flow cytometry the IEL pattern in rat small intestine during suckling (Figure 2(a)) [30]. The number of IELs in rats expands after birth, and almost all the major IEL subsets identified in adults are already present in suckling rats, but in different proportions. During the first few days of life, CD3+ CD45− IELs colonize the rat small intestine epithelium. Throughout the suckling period, there is an increase of CD3+ IELs, parallel to that observed in TCR*αβ*+cells (Figure 2(a)), the same occurs in mice [49]. Analogously, in humans, IELs increase exponentially after birth and up to 10-fold by 1-2 years of age [50].

Among neonatal intestinal IELs, NK cells are relatively abundant (Figure 2(a)) and, at weaning, their proportion is still higher than that present in adults [30, 51–53]. In addition, intraepithelial NK phenotype varies during suckling: at birth, most intraepithelial NK cells are CD8+ and, thereafter, there is an expansion of CD8- NK cells, being the main NK IEL population in adult rat. Moreover, a high proportion of intestinal intraepithelial NKT cells is found during early life and it shows a marked age-decreasing pattern being only about 1% of IELs in adult rats [53]. During the first days of rat life, besides NK cells, there is a significant proportion of TCR*γδ*+ IELs (Figure 2(a)) [30, 54], while these cells appear later in blood [55]. There is no data about NK cells in gut of human newborns; however, these cells are important in the innate responses in human adult gut [56]. On the other hand, TCR*γδ*+ cells are relatively important in human fetal IEL and decrease later [22].

The proportion of TCR*αβ*+, that mainly are CD8+ IELs, increases progressively during the suckling period in rodents [30, 54, 57] and becomes the predominant population at the end of the first week of rat life (Figure 2(a)) [30]. In contrast to peripheral cells, CD8+ IELs can be either CD8*αβ*+ (type a) or CD8*αα* (type b) [58]. CD8*αα*+ IELs develop in the gut microenvironment, can be CD4+ and either NK, TCR*αβ*+ or TCR*γδ*+, and express an oligoclonal TCR repertoire [58–61]. CD8*αα*+ IELs are thought to have an extrathymic origin or derive from early thymus precursors [62] and, in rats, are abundant cells both during suckling and adult life [30, 54]. CD8+CD4+ IELs are hardly found during the suckling period and expand after weaning (Figure 2(a)) [30, 63]. In a similar way to rat development during suckling, the TCR*αβ*+ CD8*αα*+ IELs are prevalent in the human fetal intestine initially appearing from 12 to 14 weeks of gestation and decreasing thereafter, being rather rare in adults [58].

Intestine lamina propria (LP), placed between epithelium and the *muscularis mucosa*, contains, when achieves mature state, effector cells as mature IgA-producing plasma cells, T lymphocytes (mainly Th), macrophages, dendritic cells, and mast cells [64]. In immunologically mature LP, the intestinal immune response is primarily characterized by the production of secretory IgA by plasma cells, which predominate in human intestinal LP and represent approximately 80% of all Ig-producing cells in the body [65–67]. The transport of Ig across the intestine is mediated by the polymeric Ig receptor (pIgR).

LP lymphocyte (LPL) composition in newborn rats is quite different from that in adults (Figure 2(b)). Adult rat LPLs show a predominance of CD4+ T cells, followed by B lymphocytes, a small proportion of CD8+ cells and also a minor population of NK cells (Figure 2(b)) [31, 68]. However, during the first 14 days of rat life, the proportion of CD8+ LPLs is 2–4 times higher than that of CD4+ LPLs (Figure 2(b)) and no Ig-secreting cells are present [31]. During the first week of rat life, CD8+ LPLs express the typical mucosal molecule CD8*αα* and lack the thymus-derived marker CD5. These data suggest that the subpopulation which controls the early antigen stimulus of the luminal contents is thymus-independent and is developed in the intestine LP, in a similar way to the intraepithelial compartment [31]. At the end of the second week of life, rat gut LP increases the content of CD8+CD5+ and CD8*αβ*+ cells, which seems to reflect an increasing colonization and defense provided by CD8+ cell subsets originating from the thymus [31].

Another particular lymphocyte population that seems to be relevant in LP during these first stages of development is the NK subset (Figure 2(b)). There is a relatively high percentage of NK CD8+ cells during the first two weeks of rat life, differing from adult age, when there is only a low proportion of NK LPLs and they do not bear CD8 [31]. Although little is known about the presence and phenotype of intestinal LP NK cells, this particular subset may act in the nonspecific immune response needed in the first days of life when subsets involved in specific immunity are not yet developed. During the first two weeks of rat life, there are only a few B cells in the intestinal LP (Figure 2(b)) but, at weaning, B cells become the main LPL [31] although their ability to produce antibodies is lower than in adults, and IgM-secreting cells are more abundant than those producing IgA, similar to human development (Figure 2(c)) [31]. The production of polymeric Ig receptor (pIgR), involved in the mucosal secretion of IgA and IgM starts, in rats, at the age of 19–22 days [69]. CD4+ T LPLs, the most predominant subset in adult rat LP, is the last lymphocyte population to expand and this occurs after weaning (Figure 2(b)) [31].

Gut LP development in humans shows that scattered B cells and T cells are present from 14 weeks gestation [46]. T cells in intestine LP expand during the fetal period and have a density similar to the postnatal intestine by 19–27-week gestation [46]. Plasma cells in LP do not appear until about 12 days after birth. At first, IgM cells are more common than IgA plasma cells, but by 1 to 3 months, IgA plasma cells predominate. There is a switch from monomeric IgA to polymeric IgA during the first year and, moreover, at birth, secretory IgA1 is the predominant subclass but sIgA2 increases rapidly by 6 months of age [48].

### 3.3. Biomarkers of Immune System Development in Rats

Knowledge of the development of the immune system allows the examination of the effect of particular nutrients on the time course of immune maturation and function.

Considering systemic immune system, lymphocyte composition of rat spleen during suckling shows an immature pattern that, at weaning, differs from that of adult animals. Due to the predominance of B cells during the first weeks of life, the ratio between B- and T-cell percentage could indicate the maturation phase of the spleen. In addition, the proportion of CD4+, and CD8+T cells, very low in the first days of life, also indicates the stage of the immune development of the spleen. The maturation of immune system functionality can be also studied considering the lymphoproliferative responses against specific T- and B-cell mitogens, which develop during suckling and finish later after weaning. Moreover, Th1 and Th2 responses could indicate the maturation state in young rats. These can be achieved by means of cytokines produced by isolated cells under stimulation.

With regard to the intestine compartment, there is a close association between maturation of the small intestine and activity changes of the mucosal immune system in early rat life [70]. Intestinal length and weight, enzymatic activity, crypt and villus length and width, and microbiota composition, among others, are useful tools for evaluating the primary impact of a nutrient on the maturation of the rat small intestine. In this sense, the degree of development of such variables increased during suckling without achieving adult values at weaning become a useful biomarker for evaluating the modulatory action of certain nutrients.

On the other hand, it can be studied more specific immune elements that change during suckling. In this context, IELs and LPLs are under influence of intestinal commensal bacteria, which help in the development of the immune function. Intestinal microbiota enables the IEL expansion and the acquisition of their cytotoxic ability, promotes IgA production by LPLs, and interacts with antigen presenting cells (APC) inducing the activation of regulatory cells and stimulating the tolerance against these bacteria [71].

In rats, the development of IELs after birth includes a first phase with a high content of NKT and NK cells, and furthermore these cells change from a typical systemic phenotype (CD8+) to a characteristic intestinal phenotype (CD8−). Thus, a predominance of both NK and NKT cell subpopulations in the epithelium of the small intestine is characteristic of the early life rats. Meanwhile, there is a progressive rise in acquired immunity associated with the TCR*αβ*+CD8+ IELs. Later, after weaning, IELs undergo CD8+CD4+ subset expansion [30]. Therefore, it could be interesting to establish as possible biomarkers of immune maturation in the small intestine intraepithelial compartment, the transition of NK CD8+ to NK CD8− cells, and the expansion of TCR*αβ*+ and CD8+CD4+ lymphocytes.

During the first two weeks after birth, rat LPLs development shows a predominance of CD8+ lymphocytes and NK cells. Later, around weaning, B cells expand, and afterwards and in adult life, CD4+ LPL are the most common [31]. Functionally, Ig-secreting cells are scarce during suckling and, as in humans, cells producing IgM are the most precocious. Therefore, the immune maturation of LPL could be estimated by establishing the proportions of CD4+ and CD8+ lymphocytes and B and NK cells. In addition its functionality can be measured by the number of cells producing antibodies and by the transition from predominance of IgM- to IgA-secreting cells.

## 4. Suckling Rat as a Model for Immunonutrition Studies

**Figure 3:**
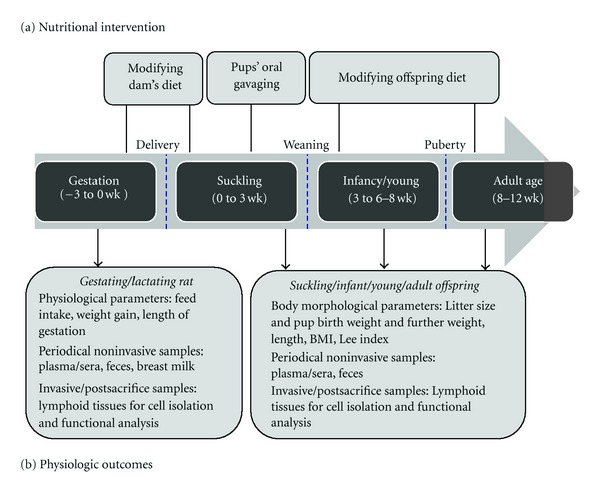
Diagram of the possible designs for experimental nutritional interventions beginning at gestation, through suckling and infancy to rat adult age and main physiological outcomes. (a) Nutritional interventions: maternal diet can be manipulated during gestation (3 wk) and/or suckling (3 wk) in order to transfer the nutrient to the offspring. During suckling, a precise amount of nutrient can be administered to pups by oral gavage. When the pups start eating the solid diet (2-3 wk), their diet can also be manipulated up to adult age. The objective of the study will lead the researcher to decide the interventional period (with the experimental diet) and the period in which the effect will be evaluated (end point of the design). (b) Physiological outcomes: samples from the gestating and lactating dams during the study period are needed to confirm nutrient incorporation and later transfer to pups (i.e., breast milk and plasma). Plasma and feces are noninvasive samples that can also be obtained periodically from the developing animals and are useful for immune determinations such as cytokines or immunoglobulins. At the end of the study, immune lymphoid tissues can be obtained for cell isolation and further phenotypic and/or functional studies (usually after *ex vivo* culture under stimulation).

The introduction of dietary supplements in strategic periods during immune system development (both systemic and intestinal) potentially allows us to identify nutrients with immunomodulatory properties and to establish when nutritional intervention can result in optimal outcomes. Studies aimed at knowing the effects of certain immunonutrients in early life can use different approaches consisting of manipulating the maternal diet during gestation and/or lactation, or feeding directly to the pups (Figure 3).

### 4.1. Nutritional Intervention on Gestating and Lactating Rats

The early gestation period of rats (days 0–7) corresponds to the embryonic phase of development because embryo implantation occurs at around day 4-5 [72]. The mid gestation period (days 8–14) largely corresponds to the time of organogenesis, while late gestation (days 15–22) is the period of the fastest growth and structural differentiation [73]. Overall, for rats, the pregnancy period lasts 21–23 days and is followed by a suckling period of about 21 days [10, 11].

A nutritional intervention can be performed during the gestation period. In this case, the breeding must take place in the own animal facilities where the researcher can control the timings and the diets of the animals [74]. In these studied animals, pregnancy should be confirmed by using, for example, sperm-positive vaginal smears. Another possibility is to obtain the gestating (G) animals as G7 or G14 from the supplier, and therefore the researcher still has 14-7 days, respectively, for the dietary intervention [75, 76]. In this case, nutritional intervention in dams determines the experimental group. When the targeted interventional period for the immunonutrition study is the suckling period, the diet can start just after the delivery up to weaning [77–79]. In this case, it is possible to randomize animals into different experimental groups on the basis of pup criteria (i.e., birth body weight). Obviously, in both designs (gestation and suckling nutritional interventions), for a better following up of the process, dams (gestating or lactating) should be housed in individual cages under controlled temperature and humidity conditions in a 12 h : 12 h light:dark cycle and with access to food and water *ad libitum*. Daily food intake during pregnancy and/or lactation and weekly body weight should be recorded [74, 76].

With regard to the dietary intervention during suckling, the target nutrient can either be directly administered by oral gavage (Section 4.2) or added to the maternal diet [74, 80, 81]. Alternatively, nursing bottles adapted to mice have been described [82] and applied in some studies [83]. The incorporation of the nutrient in the maternal diet is an easy, physiological and nonstressing way to perform the dietary intervention and is therefore fully recommended. As with any nutritional intervention, a pelleted diet containing the test nutrient should be isoenergetic in comparison with the reference diet, and its macro- and micronutrient composition as similar as possible. These diets are usually modifications of the American-Institute-of-Nutrition- (AIN-), 93 M diet, which is specially formulated for the growth, pregnancy, and lactational phases of rodents [84]. Moreover, it would be interesting to evaluate the quantity of the test nutrient in the diet. In this sense, its oxidation or degradation during the diet preparation or conservation must be avoided, and this is particularly important in the case of adding lipids as PUFAs [80]. For example, when the test nutrient is labile, it is crucial to protect the pelleted diet from light, temperature, humidity, oxygen by an appropriate packaging and changing the diet in the cage every day. It is important to bear in mind that suckling rats usually start eating the dam's pelleted diet around the second week of life (days 12–14), and therefore the intake of the test nutrient can be increased [10, 11, 79].

When the immunonutrition study focuses on the earliest lactating period, certain considerations need to be borne in mind. The delivery can be natural or induced. In this regard, some authors allow the rats to deliver at term [77, 79] and some others induce it by subcutaneous injection of oxytocin (1 IU per animal) on the 21st day of their pregnancy [85]. Variables such as litter size should be recorded in order to discard differences among groups before dietary intervention or to evidence the effects of a nutritional intervention during gestation. Precise and easy measurements in litters comprise body weight and body length (nose-anus length), which can be used to determine the body mass index (BMI), calculated as body weight/length^2^ (g/cm^2^), and Lee index, calculated as ^3^ *√*weight/length (^3^ *√*g/cm) [76].

In order to minimize variation among groups in the nutrition of the pups during suckling, litters must be pooled and redistributed to keep the same number of pups per lactating dam. Although it depends on rat strain, the number of delivered pups usually ranges from 7 to 12 [78, 86]. Litters with less than 7 or more than 12 pups should be excluded from the experiment. Groups should not be constituted by a unique dam and litter because the influence of the dam on the pups' development can mask the effect of the nutrient. Thus, at least 2-3 litters per compound should be required. Depending on the number of test nutrients or test doses and the type of determinations to be performed on the individual animals of the offspring, it may not be possible to perform the entire study at the same time, and sometimes it requires a progressive experimental design with different cohorts, including in every cohort groups representative of each condition. Moreover, due to the early age of the animals, sometimes is also needed the pooling of samples from different individuals from the same group [30, 31].

**Figure 4:**
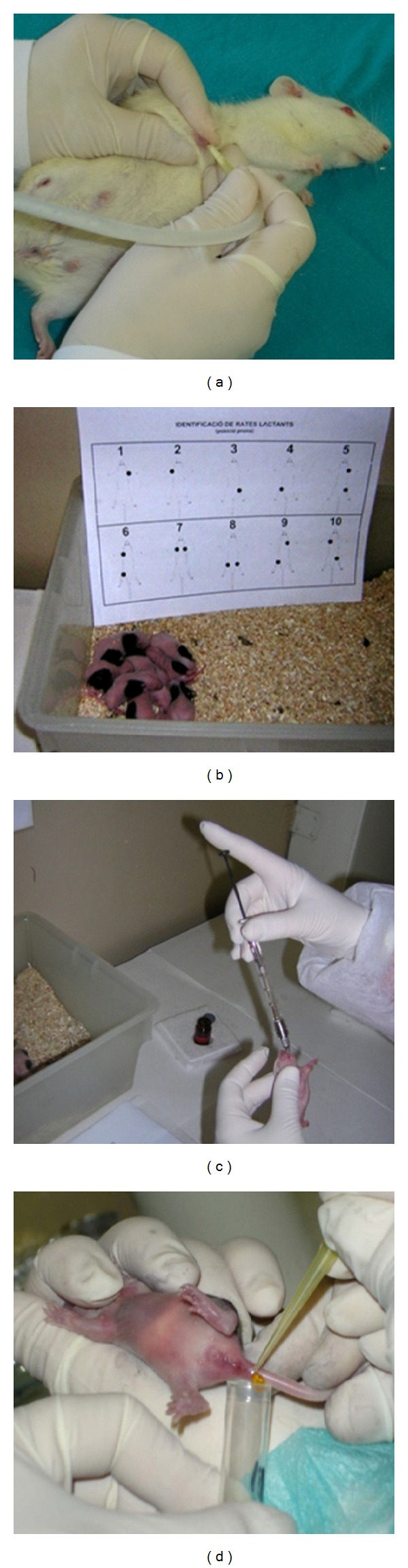
Images showing dam and offspring handling. (a) Milk from dams is collected by using elastic tubing connected to a gentle suction. (b) Animal identification by labeling the animals with a marker pen on the skin. (c) Oral gavaging in newborns younger than 5 days with ophthalmic 23-gauge gavage tubes and a short-volume syringe. (d) Example of noninvasive sampling (feces) from a neonatal rat.

When an immunonutrition study includes a protocol of feeding dams by test nutrient, the test nutrient absorption and tissue/plasma/milk incorporation by dams should be confirmed. For this purpose, maternal blood samples at different gestational/suckling ages could easily be obtained by experimental procedures performed in accordance with the institutional guidelines for the care and use of laboratory animals established by the corresponding Ethical Committee [76]. In addition, the amount of the nutrient in the offspring's blood can be and indicator of the efficiency of the transfer from the dam to the pups. Blood from animals just after birth can provide information about the transplacental transfer of the nutrient, whereas the amount of nutrient in the blood of older neonatal animals can represent the nutrient transferred either during gestation or during suckling and coming from maternal body stores. For that reason, also it is necessary to evaluate the presence of the nutrient in breast milk. Traditionally, to achieve this objective, milk from the pup's stomach has been obtained after animal sacrifice. In the last decade some groups have described how to collect and process the milk directly from the dam [76, 87, 88]. Briefly, pups are separated from dams during a certain time period (i.e., 1 h) to allow the milk to accumulate in the mammary glands. Then dams are anesthetized (e.g., intramuscularly with ketamine, 90 mg/kg rat) and then intramuscularly treated with 2–5 IU oxytocin around 10 minutes before milking. By gentle hand-stripping of teats, milk droplets can be collected in a test tube using silastic tubing connected to a gentle suction (Figure 4(a)). Depending on the type of nutrient and methodological aspects, total milk or milk whey supernatant fraction, obtained after centrifugation and fat layer discarding, can be used for nutrient quantification [76].

Some nutritional interventions mainly focused on suckling animals can also induce some effects on dams. This kind of protocol enables the study of both mother immune variables and plasma composition, which can also influence pup immune development. As an example, when a PUFA is introduced at a high proportion in the dam's diet, it is interesting to evaluate changes in fatty acid composition in the milk and plasma of the dams [76]. The changes in proportion of saturated n-3 and n-6 fatty acid patterns and plasma ratio of n-3/n-6 can be a key factor in a possible role on the immune development and passive defense of the litter in the experimental group. On the other hand, the test nutrient can also influence the formation of immune-mediators or their accumulation in the mammary gland and therefore its transfer by milk to pups. A clear example is the change in milk immunoglobulin concentration that can be induced after certain types of immunonutrients [76].

### 4.2. Nutritional Intervention on Suckling Rat: Oral Gavage

Although the dietary manipulation of the pregnant or lactating dam is a suitable approach, it is not always the best option for immunonutrition studies in early life. The test nutrient can be affected by the mother's metabolism and therefore the direct effect on the offspring could be misinterpreted. In addition, there are some studies focused on a particular nutrient present in an infant formula. In this case, the administration of the product must be performed directly in the offspring. Finally, some studies require a precise control of volume and nutrient intake. For all these reasons, and as early suckling pups do not eat a pelleted diet, oral administration to pups or even artificial rearing is alternative methodologies.

Oral gavaging of neonatal rats requires some aspects concerning handling of the litters to be taken into consideration [15]. Daily handling should be done during the same time range to avoid influences on biological rhythms. On the basis of our own experience, some actions can help to avoid the rejection and/or cannibalism from the lactating dam of handled pups, such as not wearing perfumes or strong smelling substances. Also it is recommended to allow the rat bed material to pervade the hands prior to handling the litters, or separating the mother into another cage while pups are handled.

Animal identification by labeling the animals with a tag or just with a permanent marker pen on the skin can facilitate the daily assessment of the animals (Figure 4(b)). As pups are continuously licked by their dam, when the permanent marker is used, animals have to be spotted every day.

As mentioned before in the case of the intervention through the dam's diet, in order to homogenize the litter size and weight, pups from different mothers can be mixed when they are born on the same day and before dietary intervention [77, 78]. This redistribution is limited when the diet has already been manipulated during gestation.

Dietary intervention by oral gavage has to guarantee that the volume is within appropriate limits for the size and species of the animal [13], which in the case of rats is limited to 10 mL/kg/day. The dose of the nutrient can be the same during the suckling period or can be adjusted to body weight. A fixed amount of test compound is usually used in probiotic studies [89]. But sometimes a useful approach is to adjust the amount of tested nutrient as an extrapolation of the consumption from an adult diet in which the proportion of the nutrient is known in the pelleted diet (i.e., 1% w/w). In order to calculate the equivalent volume of the solution to administer to neonates, data from the daily intake of rats from 21 to 28 days old (10–15 g chow/100 g of rat body weight) can be used [79].

The method of oral administration to baby rats needs skill and experience so as not to cause injury to their weak upper gastrointestinal tissues. As the volume to be administered is very low, a precise technique may involve using low-capacity syringes (i.e., Hamilton Bonaduz, Bonaduz, Switzerland) (Figure 4(c)). These volumetric tools should be adapted to oral gavage tubes, which can be different depending on the age of the animal. Our group, for example, is adapting them to human ophthalmic 25- or 23-gauge gavage tubes, 27 mm in length (ASICO, Westmont, IL, USA) for oral administration before and after day 5 of life, respectively [76, 77]. Oral administration can be performed several times a day without producing any adverse effects on suckling rats: no inducement of mortality, or changes in animal behavior, or interference with the dam-litter relationship, with their growth rate being similar to that of nonadministered animals. We have successfully oral-gavaged suckling rats from birth, three times a day (unpublished data), and others have even described 6 oral administrations per day [89]. It is also important to discard the influence of the oral gavaging stress or the vehicle composition and volume on the pup's development. For that reason the ideal experimental design would include, besides the group administered by oral gavage, a nonmanipulated group (reference group), a group gavaged without liquid (oral gavage stress control group), and a group receiving the same volume of the vehicle than the test nutrient group (vehicle control group).

Another aspect to be considered is that rats suckle compulsively. For this reason, in order to avoid gastric dilatation and to facilitate the introduction of the volume of test nutrient, it is recommended to allow pups to keep their stomachs empty by separating them from their dams for a brief period (i.e., 15–30 min before oral supplementation [15, 77, 78].

Finally, it is known that rats have a propensity for practicing coprophagy, a fact that can have an impact on the results of a nutritional study. In this particular context, as dams are used to eating feces from their pups during the care process, it is not suitable to mix treatments within the same litter. Each compound group has to be constituted by the whole litter.

The “pup-in-a-cup” model described in 1975 by Hall [90] for rats has since been adapted for mice, due to its primary use in transgenic research [91]. This model allows both the quantity and the composition of the dietary intake in the pups to be manipulated, by inserting an intragastric feeding tube, without the interference of the compounds present in breast milk. Some limitations of this model are that artificial rearing induces significant differences in some anatomical and physiological parameters when compared to maternally reared animals. In addition, artificially reared pups are deprived of their maternal contact, and besides the importance of this relationship for their physiological development, pups have to be hand stimulated to urinate and defecate by the researcher [91].

## 5. Nutritional Intervention after Suckling

After weaning, animals are separated by sex and the immunonutrition intervention can last until adult age [80]. Weaned animals can then consume a pelleted diet containing the test nutrient. However, it should be taken into account that the optimal diet composition for rats just after suckling, that is, during growing periods, is, among other differences, higher in protein and lipids but lower in carbohydrates (AIN-93G) than that required for the maintenance of adult animals (AIN93 M). Therefore, the basal flour in which the nutrient will be incorporated should be different depending on whether the intervention is to last until young or adult age. However, more interesting than a long-term feeding study [80] could be the impact of the dietary supplementation just during a certain critical period and to evaluate its effect later in life [92, 93]. The observation of a significant effect several weeks after finishing the dietary intervention can demonstrate the test nutrient action in the immune programming during development.

## 6. Immunomediated Pathologies in Early Rat Life

Besides the importance of studying the role of certain nutrients in the physiological development of the immune system during early life, there are pathological situations in which the addition of such compounds can help in their prevention or can contribute as curative treatments. This is the case with malnutrition or overnutrition and their impact on the immune system later in life, or common infective and inflammatory pathologies such as acute gastroenteritis in infants, necrotizing enterocolitis (NEC) or allergies and tolerance disorders, among others. Some experimental studies in different animal models, including the suckling rat model, have been performed in this regard.

### 6.1. Malnutrition/Overnutrition

Altered nutrition (i.e., malnutrition/overnutrition) during gestation or suckling may affect, among other functions, the immune system development of neonatal animals or produce a long-term impact on adult or even aged animals.

Undernutrition in pregnancy has been identified as an important risk factor of many disease states [94]. Experimentally, the intrauterine growth restriction rat models involve exposure during fetal life to relatively short periods of undernutrition either by an intrauterine artery ligation or by micronutrient deficiency or restriction of food intake, as well as by dexamethasone exposure [95]. For example, the model of low protein (LP) feeding during rat pregnancy consists of feeding gestating rats with either a control diet protein (~17–19% casein) or the deficitary diet (low protein diet, ~8-9% casein) [96]. In the case of overnutrition during gestation, a high-fat maternal diet during pregnancy, for example, with an additional 20% fat in the form of lard, has also been used as a model for developmental programming [93].

With regard to the suckling period, a protein-free diet in dams for the first 10 days of lactation causes protein malnutrition during the perinatal period in the offspring [97]. As stated before, litters from different mothers are culled to a standard number of pups to minimize variation in their nutrition during suckling. However, in order to study the malnutrition during this period, there is an experimental model which consists of using a nursing mother with a larger litter (i.e., ~15 pups/dam) (malnourished rats) and comparing it with mothers that have around 8–10 pups/dam (well-nourished rats) [45]. This model of malnutrition during suckling has shown an impairment in the development of B- and T-cell maturation in PP and MLN [45, 98].

Conversely, overnourished rats during suckling can be obtained by adjusting litter size to a smaller number of pups (i.e., 4 pups/dam) [99]. Besides this easy model, overnutrition during suckling can also be achieved by manipulating dams' diet, for example, by using diets with different fat:carbohydrate content [100] or some of the highfat diets provided by animal food companies [86]. Using both of the above models it has been demonstrated that postnatal overnutrition affects the ontogeny of intestinal microbial communities [86, 99].

### 6.2. Necrotizing Enterocolitis

Necrotizing enterocolitis (NEC) is the most common gastrointestinal disease in premature infants and is associated with high mortality and morbidity. Although mice and pig models of NEC exist, the oldest [101] and the most used are neonatal rat models which are based on damage provoked by overfeeding, pathogenic bacteria/endotoxins or stress due to hypoxia and/or hypothermia, or combinations of these [6, 102, 103]. NEC induction must be histopathologically validated by scoring the ileal tissue during the process. These models and determinations may help not only in the understanding of the protection mechanisms in the premature intestine and their contribution to NEC, but also in the modulatory action of some dietary interventions which could be added in a specific infant formula. Among the different approaches, hypoxia seems to be the crucial instigating factor [104] and nutritional interventions to ameliorate this disease are mainly based on the modulation of bacterial colonization by probiotics.

A method to develop NEC based on stress involves inducing asphyxia (breathing 100% nitrogen gas for 60 s) and cold stress (4°C for 10 min) twice daily for 4 days [105, 106]. Using this model, it has been observed that oral administration of several probiotics, such as *Bifidobacterium bifidum* or *Lactobacillus bulgaricus*, may help in the protection of the small intestine against NEC and reduce the associated inflammation in the ileum as well as the specific modulation of some molecules involved in this process [89, 107, 108].

Another approach consists of inducing NEC immediately after birth by a combination of both gavaging twice daily with a special puppy formula and inducing hypoxia 3 times daily (5% oxygen and 95% nitrogen) up to the 4th day of life [109]. For example, in this experimental NEC model in rats, a dietary intervention with resveratrol, a polyphenol compound with antioxidant and scavenger properties, modulates key enzymes in the cell cycle including iNOS and prevents mucosal damage [85].

Finally, some researchers have modified the above models by including the administration of lipopolysaccharide (LPS, 2 mg/kg) in addition to gavage feeds, hypoxia, and hypothermia [110].

Although the rodent model is not “perfect” for studying NEC pathogenesis and modulation, it seems better than the pig model, which lacks the hypoxic insult that is thought to happen in the human development of NEC [103].

### 6.3. Rotavirus Diarrhea

Acute gastroenteritis in children under two years is mostly caused by Group A rotaviruses (RV) which infect enterocytes of the small intestine and cause severe dehydration. RV diarrhea produces high worldwide morbidity, and symptomatic treatment such as rehydration is the only way to control it [111, 112].

Several animal modelsin mice, rats, and pigs have helped to advance knowledge about the infection process and pathophysiology of group A RV-associated diarrhea [112, 113]. Some of these experimental models performed in rats have been induced in germ-free suckling rats [114]. Later, Ciarlet et al. [112] developed an extensive work on the characterization of the diarrhea process in suckling rats with some heterologous RV strains. The model globally consists of inoculating orally with a high dose of the specific RV strain, usually SA-11, in early suckling life (i.e., 5–7-day-old animals) and evaluating the process by growth rate and clinical indexes based on stool appearance and consistency (Figure 4(d)). Besides these outcomes, our group has provided a self-limiting acute model that show some of the mechanisms involved in immune protection and resolution of the diarrhea process. It provides some immune response biomarkers, such as lymphocyte phenotype and proliferative ability, which may help to evaluate the activity of several food compounds, not only by shortening the diarrhea process, but also by enhancing the specific RV immune response [115].

Using these models, dietary intervention in early life with several compounds (i.e., probiotics, prebiotics, or whey protein concentrates) in RV-infected animals has been shown to provide a significant reduction in the incidence and severity of diarrhea as well as changes in viral shedding or some immune variables [116, 117]. These studies are the first step towards including such compounds in human infant formula with the objective of enhancing immune development and protecting against virulent diarrheic process.

### 6.4. Allergies and Oral Tolerance

Food allergy represents an important health concern in the Westernized world because epidemiologic data show that 6–8% of children below 3 years of age reveal food allergic reactions and about 4% of the general population are estimated to have an IgE-mediated food allergy in the United States and Europe; in addition, epidemiologic studies also evidence the increase in allergy and food allergy in industrialized countries [118]. Several experimental models in rodents have been established in an attempt to provide insights into the complex pathophysiological and immune mechanisms of human allergic diseases and asthma. At present, one of the most used animal models for evaluating food allergy is the Brown Norway rat, a high IgE responder strain. This model, which was described in detail by Knippels et al. [119], satisfies criteria of orally sensitization and challenge, with no use of adjuvant and IgE production, among others [120]. Although several dietary interventions in this model have shown their immunomodulatory activity in young-adult animals [121], very few studies have focused on earlier periods of life [122]. For example, this rat strain is also suitable for studying how diet can modulate spontaneous allergic sensitization when the early oral allergen exposure is performed during maternal milk feeding [123]. Therefore, this model can also be used to ascertain the dietary modulation of the development of oral tolerance in early life. Regarding the rat as a model for asthma, it offers some specific advantages and similarities to humans when compared to other animals, for example, due to the existence of a mucosal blood supply from the bronchial arteries, which lacks in mice [124].

## 7. Concluding Remarks

Diet plays a crucial role in maintaining optimal immune function, but, in addition, during early life it also exerts an important role in the immune development of the neonate. Due to the limited data from epidemiological and human interventional trials in early life, animal models hold the key to increasing the current knowledge about the nutrition-immunity interaction in this particular period. However, which experimental model is the most appropriate? We think that two aspects are crucial when choosing the animal model for an experimental design: the experimental feasibility of the dietary intervention and knowing which immune biomarkers can allow us to examine whether the supplementation with the nutrient of interest accelerates its physiological time course maturation. The suckling rat immunonutrition model presented here satisfies both aspects.

Some authors think that short-gestation-period animals, such as rats, which are born with a very immature physiology (i.e., gastrointestinal and immune systems), are less suitable than other models based on longer-gestation animals such as the pig, which is more similar to humans at birth. However, in our opinion, the rat model has some advantages due to these short periods and immaturity. In this sense, the interventional procedure can be more easily performed over a whole period (i.e., gestation and/or suckling) in a relatively short time and with a higher cost-effective ratio due to the intrinsic characteristics of rat physiology. In this paper we have provided some methodological aspects to bear in mind for the experimental design. Furthermore, the anatomy and immune function of the rodent gut are immature at birth but develop rapidly during suckling and throughout weaning. This postnatal period, continuously changing, is optimal for performing the dietary intervention and evaluating whether the test nutrient modulates the immune biomarkers (i.e., proportions of immature cells, ability to proliferate or to synthesize immunoglobulins) to a more mature proportion or activity, which have to be more similar to that found in adult age.

It is true that the extrapolation of data obtained from rodents to humans should be carefully evaluated due to physiological differences, but in the field of nutritional modulation of the immune system in early life, there are relatively few studies justifying a specific animal model.

## Figures and Tables

**Table 1:** Physiological characteristics and logistical considerations of the three most used animal models for nutritional intervention in early life [10–15].

	Mouse	Rat	Pig
Physiological characteristics			

Pregnancy period (days)	18–21	21–23	110–118
Placenta type	Discoidal, hemoendothelial choroidea, decidual	Discoidal, hemoendothelial choroidea, decidual	Epitheliochorial
Litter size	6–12	6–15	11–16
Birth weight (g)	0.5–1.5	3–5	900–1600
Weaning weight male/female (g)	18–25/16–25	55–90/45–80	6000–8000
Suckling period (days)	21–28	21	28–49
Solid diet beginning (days)	10	12	12–15
Puberty male/female (wk)	4–6/5	6/6–8	20–28
Life expectancy (years)	1-2	2-3	14–18
Developmental maturity at birth^1^	∗	∗	∗∗

Animal model logistical considerations^2^			

Minimum enclosure size for mother and litter (cm^2^)	330	800	0.2–2.5 m^2^
Purchase and maintenance cost	∗-∗∗^3^	∗	∗∗∗
Availability	∗-∗∗∗∗∗^3^	∗∗∗∗∗	∗∗∗
Easily of pup's manageability	∗∗	∗∗∗∗∗	∗∗∗

^1^Developmental maturity (i.e., gastrointestinal functions—nutrition and evacuation—thermoregulation, locomotion, etc.) related to adults from immature (∗) to mature (∗∗∗∗∗).

^2^Animal model desirability factors evaluated from relatively low (∗) to relatively high (∗∗∗∗∗).

^3^Cost and availability differ depending on the particularities of the animal strain.
